# THE EFFECTS OF AGE AND SEX ON THE GUT MICROBIOME AND GUT HOMEOSTASIS IN MICE

**DOI:** 10.1093/geroni/igad104.0293

**Published:** 2023-12-21

**Authors:** Jennifer Lee, Kerry Wellenstein, Gregory Matuszek

**Affiliations:** Tufts University, Boston, Massachusetts, United States; Tufts University, Boston, Massachusetts, United States; Tufts University, Boston, Massachusetts, United States

## Abstract

Symbiotic interactions between the gut microbiome and intestinal epithelium are key contributors to regulating gut homeostasis and host metabolism in mice. However, the majority of studies measuring these outcomes have been reported in male and not female mice. Here, we find sex-specific differences in gut homeostasis and host metabolism in conventional C57BL6 chow-fed mice. Functional analyses of the gut metagenomes and metabolomes, gut barrier function, and colonic T-cell populations are different between young and old female and male mice. These data highlight the importance of considering sex as a biological variable in aging rodent studies, which may lead to optimized strategies to improve healthy aging for all.

